# Association of source country gender inequality with experiencing assault and poor mental health among young female immigrants to Ontario, Canada

**DOI:** 10.1186/s12889-021-10720-0

**Published:** 2021-04-16

**Authors:** Michael Lebenbaum, Therese A. Stukel, Natasha Ruth Saunders, Hong Lu, Marcelo Urquia, Paul Kurdyak, Astrid Guttmann

**Affiliations:** 1 ICES, 2075 Bayview Ave, Toronto, ON M4N 3M5 Canada; 2 Institute of Health Policy, Management and Evaluation, University of Toronto, 155 College St 4th Floor, Toronto, ON M5T 3M6 Canada; 3 Division of Paediatric Medicine, the Hospital for Sick Children, 555 University Ave, Toronto, ON M5G 1X8 Canada; 4 Department of Pediatrics, University of Toronto, 555 University Avenue, Black Wing Room 1436, Toronto, ON M5G 1X8 Canada; 5 Child Health Evaluative Sciences, SickKids Research Institute, 686 Bay St, ON M5G 0A4 Toronto, Canada; 6 Dalla Lana School of Public Health, University of Toronto, 155 College St Room 500, Toronto, ON M5T 3M7 Canada; 7 Manitoba Centre for Health Policy, Community Health Sciences, Max Rady College of Medicine, 424 Brodie Centre, Winnipeg, Manitoba Canada; 8 Rady Faculty of Health Sciences, University of Manitoba, 424 Brodie Centre, Winnipeg, Manitoba Canada; 9 Center for Addiction and Mental Health, 33 Russell St, Toronto, ON M5S 3M1 Canada

## Abstract

**Background:**

Gender inequality varies across countries and is associated with poor outcomes including violence against women and depression. Little is known about the relationship of source county gender inequality and poor health outcomes in female immigrants.

**Methods:**

We used administrative databases to conduct a cohort study of 299,228 female immigrants ages 6–29 years becoming permanent residence in Ontario, Canada between 2003 and 2017 and followed up to March 31, 2020 for severe presentations of suffering assault, and selected mental health disorders (mood or anxiety, self-harm) as measured by hospital visits or death. Poisson regression examined the influence of source-country Gender Inequality Index (GII) quartile (Q) accounting for individual and country level characteristics.

**Results:**

Immigrants from countries with the highest gender inequality (GII Q4) accounted for 40% of the sample, of whom 83% were from South Asia (SA) or Sub-Saharan Africa (SSA). The overall rate of assault was 10.9/10,000 person years (PY) while the rate of the poor mental health outcome was 77.5/10,000 PY. Both GII Q2 (Incident Rate Ratio (IRR): 1.48, 95% Confidence Interval (CI): 1.08, 2.01) and GII Q4 (IRR: 1.58, 95%CI: 1.08, 2.31) were significantly associated with experiencing assault but not with poor mental health. For females from countries with the highest gender inequality, there were significant regional differences in rates of assault, with SSA migrants experiencing high rates compared with those from SA. Relative to economic immigrants, refugees were at increased risk of sustaining assaults (IRR: 2.96, 95%CI: 2.32, 3.76) and poor mental health (IRR: 1.73, 95%CI: 1.50, 2.01). Higher educational attainment (bachelor’s degree or higher) at immigration was protective (assaults IRR: 0.64, 95%CI: 0.51, 0.80; poor mental health IRR: 0.69, 95% CI: 0.60, 0.80).

**Conclusion:**

Source country gender inequality is not consistently associated with post-migration violence against women or severe depression, anxiety and self-harm in Ontario, Canada. Community-based research and intervention to address the documented socio-demographic disparities in outcomes of female immigrants is needed.

**Supplementary Information:**

The online version contains supplementary material available at 10.1186/s12889-021-10720-0.

## Introduction

Gender inequality remains a significant global problem limiting human development, economic growth, and health across the world [1] and among women, contributes to a lack of economic resources and power [2, 3]. Greater gender inequality measured regionally has been shown to be associated with depressive symptoms among American mothers across US states [4] and with an increase in the excess burden of depression among women in a sample of 122 countries from all world regions [5]. Research from a sample of 25 European countries suggests that gender inequality may have negative effects on depressive symptoms for both women and men [6]. Furthermore, gender inequalities are a significant contributing factor to the large global burden of violence against women [7] in both developing [3] and developed [8] countries.

Worldwide, there has been migration of large numbers of immigrants from regions with high levels of gender inequality to those with lower inequality [9]. Canada receives a large proportion of immigrants from regions with high gender inequality including South Asia (e.g., India, Pakistan, Bangladesh) and Sub-Saharan Africa (e.g., Nigeria, Ethiopia, Somalia). The Gender Inequality Index (GII) is a useful measure of country level inequality, taking into account reproductive health and inequality in economic (i.e., employment and education) and political (i.e., political representation) domains [1]. Norms and attitudes towards gender inequality among immigrants are associated with source country gender inequality/norms [10, 11] and have been shown to persist post-migration or be higher among first-generation immigrant populations [10–12]. These include attitudes and norms about the prioritization of education and employment for men and measures of acceptability of violence towards women [10–12]. Studies have found a negative association between greater source country gender inequality (i.e., female-male labour activity and secondary education ratio), and female immigrant work hours in Canada as well as a greater burden of household responsibilities [13]. Other evidence of the persistence of gender-based norms includes male-biased sex ratios in certain selected groups of immigrants to Canada from regions in South Asia with high levels of gender inequality [14, 15]. The persistence of gender inequalities post-migration may increase risk of poor health outcomes among migrant women. Only one study has directly assessed the influence of source country gender inequality and post-migration experiences of violence (as measured by intimate partner violence [IPV]) across many (41) source countries [16]. The findings indicated that greater gender equality as measured by the Gender Gap Index was associated with lower post-migration physical IPV, while high source country inequality as measured by GII was associated with greater levels of IPV [16]. No studies have systematically examined at the population-level whether variations in source country gender inequalities are associated with post-migration mental health. These findings could shed light on areas for public health interventions and resettlement services and help identify and prioritize populations for prevention.

The objectives of this study were to examine the association between source country gender inequality and the rate of severe presentations for assault and selected mental health disorders in young female immigrants to Ontario, Canada. We also explore the association between other important sociodemographic characteristics including refugee status, neighbourhood income, education, and marital status. We hypothesized that source country gender inequality would be positively associated with assaults and poor mental health outcomes and that other socio-demographic characteristics would be important. We did not have prior hypotheses that these socio-demographic characteristics would be differentially associated with outcomes by source country gender inequality.

## Methods

### Data sources

We linked and analyzed data at ICES for this population-based cohort study. ICES is an independent, non-profit research institute whose legal status under Ontario’s health information privacy law allows it to collect and analyze health care and demographic data, without consent, for health system evaluation and improvement. Individual records were linked using unique encoded identifiers across Ontario immigration, healthcare and vital statistics databases and analyzed at ICES. The Immigration, Refugees and Citizenship Canada Permanent Resident (IRCC-PR) database included all immigrants to Ontario at the point of receiving permanent residence. Administrative databases included the Discharge Abstract Database (DAD) for hospital admissions and Ontario Mental Health Reporting System (OMHRS) for admissions to mental health hospital beds (2006 and onwards); the National Ambulatory Care Reporting System (NACRS) for emergency department (ED) visits; and the Registered Persons Database (RPDB) and Census data for demographic information. Vital statistics databases included RDPB to capture death and Ontario Registrar General Vital Statistics Database (ORG-VSD) to measure death and cause of death. The country specific Gender Inequality Index (GII) were obtained from United Nations Development Programme, while Gross National Income (GNI) was obtained from the World Bank.

### Study population

The cohort consisted of female immigrants aged 6 to 29 years old at the time they obtained permanent resident status between January 1, 2003 to May 31, 2017. Sex – male or female – is determined based on demographic information available in the administrative databases. In almost all cases, sex is collected as a binary male/female variable, which is not inclusive of intersex people, and is most often the sex assigned at birth. Younger immigrants were studied since they are more likely to acculturate quickly, and risk factors for study outcomes are different in older ages. The date of entry into the cohort (index date) was that of healthcare eligibility, typically within 3 months of arrival in Canada (immediate for resettled refugees), and age 12 for those younger than 12 years at arrival. We excluded immigrants with invalid health card identifiers, as they are required to link across databases. We also excluded individuals who migrated out of Ontario or died prior to age 12.

### Study exposure groups

We used GII to measure the level of gender inequality of the source country [1]. GII measures inequalities in three key areas of human development including reproductive health (maternal mortality ratio and adolescent birth rate), empowerment (education and share of parliamentary seats), and labour (labour market participation rates) [1]. The overall GII index ranges between 0, perfect equality in all dimensions, and 1, perfect inequality [1]. We used GII data from 2000, 2005, and yearly from 2010 to 2017 [1]. We assigned each immigrant her source country GII value and World Bank GNI category (i.e., low, lower-middle, upper-middle, high-income countries) using the year closest to when she received permanent residency within 2 years. For missing GII values we used a combination of the GNI categories and 18 region of birth categories according to the Standard Classification of Countries and Areas of Interest to conduct a single imputation, given the percentage with missing data was small (6.4%) and the imputation regression model had a very high R^2^ (0.81). We categorized GII into year-specific quartiles based on balancing the number of countries across quartiles.

### Study outcomes

The primary outcomes were assaults and a composite mental health outcome, both of which were operationalized as the need for acute medical care or death. Experiencing assault consisted of ED visits, hospital admissions, or death by assault (including intimate partner violence). These ED visits and hospitalizations represent primarily physical assault and to a lesser extent sexual assault but there is no information on the perpetrator. A composite outcome of acute and severe mental health problems was defined by ED visits or hospital admissions for mood disorders, anxiety disorders, or deliberate self-harm, or suicide. While previous literature has focused on depression [4-5] , we also included anxiety as this is associated with adverse social circumstances. Other injuries, including unintentional injuries and those of undetermined intent, and all-cause mortality were secondary outcomes given under-reporting of intentional injuries [17] and suicide [18]. Diagnostic codes and databases used are provided in Supplementary Table 1. Study outcomes were assessed between the index date and end of follow-up, which included migration out of Ontario, death, or the maximum follow-up date. Due to data availability, the maximum follow-up date was December 31, 2017 for cause-specific death (homicide and suicides) and March 31, 2020 for all other outcomes.

### Covariates

Characteristics of immigrants were assessed at the date of becoming a  permanent resident. Covariates included age group, neighborhood income quintile, visa class, immigration period, level of education, marital status, and source country level of development. Neighborhood income was assessed at the level of census dissemination area (i.e. 400–700 people). Visa classes were economic (based on skills and potential economic productivity), family (sponsored family members), or refugee (resettled or successful asylum claimants) [19]. Education and marital status were measured for adults age 18 and older at time of immigration. We used the per-capita GNI categorized into year-specific levels of development according to the World Bank [20]. We also characterized countries according to World Bank regions [21].

### Statistical analysis

We used Poisson regression models to determine the association between each outcome and source country GII, accounting for differential follow-up. GII was analyzed first as a continuous measure to assess overall trends, then as quartiles to assess whether the highest quartile differed from lower ones. All analyses used the individual immigrant as the unit of analysis, and robust variances to account for clustering of individuals within source country. Due to the very large numbers of immigrants from India, China, Pakistan and Philippines, we mitigated these countries’ influence in the regression models by randomly selecting 9100 individuals from each country, producing a sample size equivalent to the fifth largest source country (i.e., Sri Lanka). Stratified random sampling with proportional sampling was used to ensure the same distribution of covariates for each country in the random and original sample. We included indicator variables for these four countries in the models since bivariate plots showed them to be outliers in terms of the relationship of GII to outcomes. Models included GII quartile, four large country-specific terms, age category, income quintile, immigration class, immigration period, and GNI category. Subgroup analyses among those age 18 years and over  at immigration further explored the role of post secondary educational attainment and marital status. As most covariates were available only at the time of immigration, causal pathway models or mediation analyses would not have been supported by the available data. Analyses were conducted in SAS Enterprise Guide version 7.1 and Stata vs. 15.1.

## Results

A total of 318,372 immigrants were eligible for inclusion in the study. Of 19,184 individuals with missing GII, we imputed GII for 17,833 respondents from 52 countries based on region and GNI category. After exclusion criteria were applied, 299,228 individuals remained with an average follow-up of 9.6 years (Supplementary Fig. 1). Otherwise, 3161 (1.5%) of subjects age 18+ who were missing data on education and marital status were dropped from a sensitivity analysis. After stratified random sampling of 9100 individuals from the largest 4 countries source countries, 204,124 individuals were included in the primary regression models and 134,338 individuals in the sensitivity analyses of individuals age 18–29. A total of 192 countries were represented in the sample with nearly all levels of gender inequality (Supplementary Fig. 2).

Immigrants from countries with the lowest levels of gender inequality (GII Q1) immigrated largely from the East Asian and Pacific region (70%) and European and Central Asian countries (21.6%). Those from countries with the highest levels of gender inequality (GII Q4) migrated largely from South Asia (67.1%) and Sub-Saharan Africa (15.7%) (Table 1). Immigrants from India and Pakistan represented 57.8% of all immigrants from GII Q4. There were important differences in socio-demographic characteristics at the time of immigration to Canada by source country GII. A higher percent of immigrants from countries with high GII were refugees, had lower educational attainment, were single, and lived in low-income neighbourhoods.

**Table 1:** Descriptive statistics for each covariate by quartile of Gender Inequality Index (GII)^a^

	Total Person Years	GII Q1 (*N* = 64,089)		GII Q2 (*N* = 38,778)		GII Q3 (*N* = 77,482)		GII Q4 (*N* = 118,879)	
		N	%	N	%	N	%	N	%
*Age at immigration (years)*									
6–9	217,151.2	5799	9.0	4115	10.6	7718	10.0	11,745	9.9
10–13	305,325.4	5824	9.1	3988	10.3	10,263	13.2	11,806	9.9
14–17	336,155.5	6544	10.2	4137	10.7	11,106	14.3	11,547	9.7
18–21	428,999.5	6607	10.3	5387	13.9	12,246	15.8	17,279	14.5
22–25	686,966.7	14,994	23.4	9184	23.7	15,011	19.4	29,994	25.2
26–29	885,133.3	24,321	37.9	11,967	30.9	21,138	27.3	36,508	30.7
*Immigrant Status*									
Economic Class	1,149,898	31,326	48.9	13,788	35.6	33,902	43.8	46,086	38.8
Family Class	1,300,666.0	28,391	44.3	20,289	52.3	32,173	41.5	48,077	40.4
Refugee	409,167.5	4372	6.8	4701	12.1	11,407	14.7	24,716	20.8
*Income Quintile*									
Income Quintile 1 (Lowest)	1,086,293.8	17,730	27.6	13,874	35.8	30,617	39.5	51,669	43.4
Income Quintile 2	682,299.9	17,171	26.8	8452	21.8	17,996	23.2	27,107	22.8
Income Quintile 3	493,501.4	11,165	17.4	6517	16.8	13,128	16.9	20,394	17.2
Income Quintile 4	362,063.4	9758	15.2	5638	14.5	9446	12.2	13,102	11.0
Income Quintile 5 (Highest)	235,573.0	8265	12.9	4297	11.1	6295	8.1	6607	5.6
*Immigration Period*									
2003–2005	1,043,228.0	17,719	27.6	9932	25.6	17,339	22.4	30,533	25.7
2006–2008	801,849.2	16,180	25.2	9383	24.2	19,571	25.3	25,299	21.3
2009–2011	525,019.5	12,122	18.9	7881	20.3	17,441	22.5	23,299	19.6
2012–2014	325,091.6	10,558	16.5	7190	18.5	12,333	15.9	22,124	18.6
2015–2017	164,543.2	7510	11.7	4392	11.3	10,798	13.9	17,624	14.8
*Region of Birth*									
East Asia and Pacific	746,448.7	44,884	70.0	5592	14.4	28,210	36.4	45	0
Europe and Central Asia	320,881.3	13,836	21.6	15,461	39.9	1817	2.3	141	0.1
Latin America and the Caribbean	315,510.1	0	0	7920	20.4	22,539	29.1	1717	1.4
Middle East and North Africa	329,639.2	2749	4.3	3976	10.3	12,930	16.7	18,441	15.5
North America	67,242.8	2620	4.1	5231	13.5	0	0	0	0
South Asia	889,515.9	0	0	0	0	10,514	13.6	79,814	67.1
Sub-Saharan Africa	190,493.5	0	0	598	1.5	1472	1.9	18,721	15.7
*Gross National Income (GNI) Categorization*									
Low-income economies	706,343.2	0	0	2755	7.1	3414	4.4	55,222	46.5
Lower-middle-income economies	1,362,576	22,101	34.5	11,344	29.3	56,206	72.5	55,147	46.4
Upper-middle-income economies	467,041.2	18,730	29.2	14,754	38.0	17,707	22.9	5661	4.8
High-income economies	323,771.6	23,258	36.3	9925	25.6	155	0.2	2849	2.4
*Marital Status (Immigrants age 18–29 at immigration)*									
Married	690,963.9	18,871	41.1	8304	31.3	22,981	47.5	24,554	29.3
Separated/widowed/divorced	12,592.48	139	0.3	253	1.0	302	0.6	559	0.7
Single	1,297,079.0	26,902	58.6	17,970	67.7	25,091	51.8	58,611	70.0
*Education (Immigrants age 18–29 at immigration)*									
Bachelors or higher	762,473.4	17,755	38.7	9263	34.9	15,221	31.5	37,165	44.4
Postgraduate less than Bachelors	388,378.9	12,661	27.6	5758	21.7	9985	20.6	10,277	12.3
Secondary or less	837,774.9	14,610	31.8	11,178	42.1	22,830	47.2	34,871	41.6

Legend: ^a^Table 1 uses full sample before stratified random sampling is applied

The overall rate of assault was 10.9/10,000 person years (PY) and the rate of the poor mental health outcome was 77.5/10,000 PY. Crude rates of experiencing assault or the composite mental health outcome were greatest in migrants from countries in the two middle GII quartiles (Table 2). Immigrants from Sub-Saharan Africa (SSA), Latin America and the Caribbean (LAC) had the highest rate of experiencing assault (SSA: 24.9/10,000 PYs; LAC: 23.7/10,000 PYs), and among the highest rates of acute care visits for mental health (SSA: 103.7/10,000 PYs; LAC: 116.7/10,000 PYs). South Asian (SA) and East Asian (EA) immigrants had among the lowest rates of assaults (SA: 6.3/10,000 PYs; EA: 7.2/10,000 PYs) and mental health acute care visits (SA: 68.0/10,000 PYs; EA: 47.9/10,000 PYs). Immigrants from China, India and Philippines had much lower rates of the mental health composite outcome relative to their GII quartile, and immigrants from India, Pakistan and Philippines had lower rates of sustaining assault relative to their GII quartiles, suggesting they are outliers compared to their GII peers. Crude rates of both outcomes were higher in refugees, separated/widowed/divorced individuals, and lower in those with a bachelor’s degree or higher. Crude rates for the sensitivity analysis outcomes including other injuries and death are presented in supplementary Table 2.

**Table 2:** Number and rate^a^ of violent injuries and mental health composite outcome^a^

	Experiencing Assault		Mental Health Composite	
	N	Rate^b^	N	Rate^b^
*Gender Inequality Index (GII) Quartile*				
GII Q1 (Lowest Inequality)	509	8.0	3895	61.5
GII Q2	543	14.3	3291	86.5
GII Q3	973	13.0	6713	90.0
GII Q4 (Highest Inequality)	1087	9.9	8272	75.2
*Largest 4 Countries*				
China (GII = 0.20; Q1)	263	7.2	1130	30.9
Philippines (GII = 0.45; Q3)	160	7.2	1499	67.9
Pakistan (GII = 0.59; Q4)	94	4.3	1571	72.3
India (GII = 0.60; Q4)	232	5.1	2140	46.8
*Age at immigration (Years)*				
6–9	227	10.5	2678	123.3
10–13	439	14.4	3398	111.3
14–17	561	16.7	3547	105.5
18–21	596	13.9	3656	85.2
22–25	638	9.3	4322	62.9
26–29	651	7.4	4570	51.6
*Immigrant Status*				
Economic	686	6.0	7127	62.0
Family/other/missing	1500	11.5	9936	76.4
Refugee	926	22.6	5108	124.8
*Income Quintile*				
Income Quintile 1 (Lowest)	1415	13.0	9035	83.2
Income Quintile 2	684	10.0	4940	72.4
Income Quintile 3	478	9.7	3742	75.8
Income Quintile 4	333	9.2	2545	70.3
Income Quintile 5 (Highest)	202	8.6	1909	81.0
*Immigration Period*				
2003–2005	1035	9.9	7571	72.6
2006–2008	884	11.0	6155	76.8
2009–2011	622	11.8	4262	81.2
2012–2014	411	12.6	2691	82.8
2015–2017	160	9.7	1492	90.7
*Region of Birth*				
East Asia and Pacific	535	7.2	3579	47.9
Europe and Central Asia	356	11.1	3120	97.2
Latin America and the Caribbean	747	23.7	3682	116.7
Middle East and North Africa	354	10.7	2894	87.8
North America	83	12.3	871	129.5
South Asia	563	6.3	6050	68.0
Sub-Saharan Africa	474	24.9	1975	103.7
*Gross National Income (GNI) Categorization*				
Low-income	792	11.2	5530	78.3
Lower-middle-income	1235	9.1	9195	67.5
Upper-middle-income	723	15.5	4132	88.5
High-income	362	11.2	3314	102.4
*Marital Status (Immigrants age 18–29 at immigration)*				
Married	850	12.3	4940	71.5
Separated/widowed/divorced	26	20.6	170	135.0
Single	1008	7.8	7434	57.3
*Education (Immigrants age 18–29 at immigration)*				
Bachelors or higher	404	5.3	3266	42.8
Postgraduate less than Bachelors	402	10.4	2337	60.2
Secondary or less	1068	12.7	6868	82.0

Legend: ^a^Table 2 uses full sample before stratified random sampling is applied; ^b^per 10,000 Person Years (PYs)

**Fig. 1:**
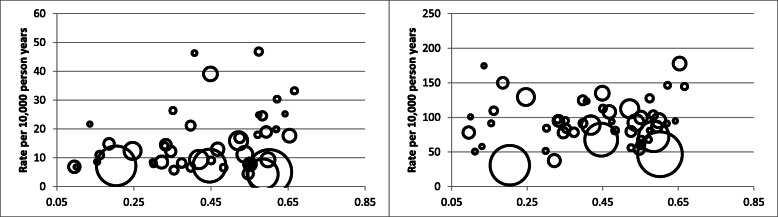
Bubble plots of continuous GII and (**a**) Experiencing Assault and the (**b**) Mental Health Composite Outcome*. Legend: *Four largest source countries: China (GII = 0.20), Philippines (GII = 0.45), Pakistan (GII = 0.59), India (GII = 0.60); Figure is restricted to the largest 50 source countries; Circle size weighted by the number of individuals from that country; 4 countries are not shown for violent injuries as they were censored due to small cell restrictions to preserve anonymity

Bubble plots demonstrating the relationship between GII as a continuous variable and both primary outcomes for the 50 largest source countries are presented in Fig. 1.

In adjusted models, there was no overall trend in rates of experiencing assault with respect to continuous GII (Incident Rate Ratio (IRR): 1.08; 95% confidence interval (CI) 1.00, 1.17; *p* = 0.063). However, immigrants in the highest compared to the lowest GII quartile had a higher rates of experiencing assault (IRR: 1.58; 95% CI 1.08, 2.31) (Table 3). Similarly, individuals in GII quartile 2 (second lowest quartile) had greater rates (IRR: 1.48; 95% CI 1.08, 2.01), while the rates for those from countries in GII quartile 3 was not significantly elevated (IRR: 1.53; 95% CI 0.99, 2.34). There was no significant association between GII either as a categorical or continuous variable and the mental health composite outcome. Immigrants from India and Pakistan had much lower rates of experiencing assault (IRR range: 0.40–0.44) and those from India, Pakistan, and China had lower rates of the mental health composite outcome (IRR range: 0.39–0.72) compared to countries in their GII quartile (Table 3).

**Table 3:** Poisson regression for association between GII^a^ and primary outcomes (*N* = 204,124)^a,b^

	Experiencing Assault			Mental Health Composite		
	IRR	95%LCL	95%UCL	IRR	95%LCL	95%UCL
*GII Quartile (Ref: GII Q1)*^*a*^						
GII Q2	1.48	1.08	2.01	0.83	0.69	1.01
GII Q3	1.53	0.99	2.34	0.92	0.73	1.16
GII Q4	1.58	1.08	2.31	0.91	0.68	1.22
*Largest 4 Countries*						
China	1.15	0.85	1.57	0.39	0.32	0.48
Philippines	0.91	0.64	1.30	0.86	0.74	1.00
Pakistan	0.40	0.33	0.48	0.72	0.62	0.84
India	0.44	0.36	0.53	0.62	0.53	0.73
*Age at Immigration (Years) (Ref:* 22–25)						
6–9	1.69	1.24	2.31	2.41	2.01	2.88
10–13	2.05	1.66	2.55	2.02	1.76	2.31
14–17	2.29	1.85	2.84	1.97	1.75	2.20
18–21	1.72	1.43	2.06	1.55	1.40	1.72
22–25	1.14	0.96	1.36	1.18	1.05	1.31
*Income Quintile (Ref: Quintile1)*						
Income Quintile 2	0.84	0.75	0.94	0.92	0.85	0.99
Income Quintile 3	0.85	0.74	0.99	0.99	0.89	1.09
Income Quintile 4	0.82	0.71	0.96	0.90	0.82	0.99
Income Quintile 5 (Highest)	0.75	0.59	0.95	1.03	0.88	1.22
*Gross National Income (GNI) Categorization (Ref: Low income)*						
Lower-middle-income	0.85	0.62	1.18	0.94	0.76	1.16
Upper-middle-income	1.25	0.87	1.80	1.10	0.85	1.42
High-income	1.21	0.82	1.81	1.07	0.77	1.49
*Immigration Status (Ref: Economic)*						
Refugee	2.96	2.32	3.76	1.73	1.50	2.01
Family	2.26	1.81	2.83	1.49	1.31	1.71
*Immigration Period (Ref: 2003–2005)*						
2006–2008	1.00	0.88	1.14	1.02	0.95	1.09
2009–2011	1.04	0.90	1.20	1.09	0.98	1.21
2012–2014	1.08	0.88	1.32	1.13	1.00	1.28
2015–2017	0.84	0.64	1.11	1.25	1.05	1.48

^a^GII = Gender Inequality Index

^b^Experiencing assault and mental health composite are modeled in separate regression models

Multivariable models assessing potential underreporting of experiencing assault showed no significant association between GII and other injuries or death (Supplementary Table 3). Among immigrants age 18–29 at time of immigration, we found higher rates of sustaining assault (IRR: 1.59; 95% CI: 1.04, 2.43) in the highest versus lowest GII quartiles, but no significant relationship between GII quartile and other outcomes (Supplementary Table 4).

Socio-demographic characteristics were associated with most of the primary and secondary outcomes. Relative to economic migrants, refugees had higher rates of experiencing assault (IRR: 2.96; 2.32, 3.76), poor mental health (IRR: 1.73; 1.50, 2.01) (Table 3), other injuries (IRR: 1.58; 95% CI: 1.47, 1.70) and death (IRR: 1.38; 95% CI: 1.03, 1.84) (Supplementary Table 3). Younger age at immigration, particularly ages 6–21, was associated with 1.7 to 2.3-fold higher rates of sustaining assault, 1.5 to 2.4-fold higher rates of the mental health composite (Table 3), and 1.3 to 1.4-fold higher rates of other injuries (Supplemental Table 3) compared to those aged 26–29. Higher neighbourhood income was associated with 25% lower rates of experiencing assault (Table 3). In the subgroup of those 18 years of age and over at immigration, having a Bachelor’s degree or higher was associated with lower rates of experiencing assault (IRR: 0.64; 95% CI: 0.51, 0.80), poor mental health events (IRR: 0.69; 95% CI: 0.60, 0.80), other injuries (IRR: 0.84; 95% CI: 0.78, 0.90) and death (IRR: 0.65; 95% CI: 0.45, 0.95) (Supplementary Table 4). Being married at the time of immigration was associated with lower rates of experiencing assault (IRR: 0.71; 95% CI: 0.62, 0.82), poor mental health events (IRR: 0.89; 95% CI: 0.80, 0.99), and other injuries (IRR: 0.90; 95% CI: 0.86, 0.94).

## Discussion

In this large, population-based longitudinal study, we found that female immigrants from some, but not all, regions with the highest levels of gender inequality had greater rates of experiencing assault compared with those from source countries with the lowest gender inequality. This relationship was not consistent across all levels of gender inequality and did not follow a dose-response trend. We did not find an association between source country GII and severe mood and anxiety disorders, or deliberate self-injury including suicide. Refugee status, lower educational attainment and low neighbourhood income were associated with greater rates of experiencing assault, poor mental health outcomes, and other injuries. For females who immigrated as adults, being married at the time of immigration was associated with lower rates of poor mental health, experiencing assault and other injuries.

The higher rates of experiencing assault among the highest quartile of GII reflects higher rates among immigrants from countries primarily in Sub-Saharan Africa, the Middle East and North Africa, with the removal of the influence of India and Pakistan in the models. Immigrants from India and Pakistan, two countries in the highest gender inequality quartile had much lower rates of experiencing assault than their GII country peers. Prior studies that have examined violence against female immigrants have tended to examine self-reported IPV [26], including one study on the influence of source country gender inequality [16] and others conducted among samples of immigrants from high gender inequality countries [26–28]. IPV may include physical, emotional, and sexual violence. Our study primarily measured physical violence and to a lesser extent sexual assault and abuse/maltreatment leading to injury severe enough to require a visit to the emergency department or hospital admission. This may underestimate the burden of assault in this population but measures the more severe end of the spectrum of assault. The results in immigrants in the highest gender inequality quartile from outside of South Asia, especially Sub-Saharan Africa, are in line with a European study demonstrating a positive association between greater source country gender inequality and higher post-migration physical IPV [16]. However, our results in some specific sub-groups, notably those from South Asia are in contrast to some of these studies that have found high levels of IPV against South Asian women in developed countries [26, 29] but not others [28].

The broader literature of violence against immigrants provides context for some of our findings. These include studies that have found bullying and other forms of peer violence to be elevated among first generation immigrants from non-English speaking countries [22]. Ethnic, racial and religious discrimination are commonly reported by racialized immigrant groups in Canada [23] and is associated with a greater risk of experiencing assault [24, 25], including hate crimes [30]. Our findings of higher rates of assault among refugees is consistent with prior research in Ontario [31]. The large negative relationship between education and assaults is in keeping with prior research examining family violence and violence against women in immigrant populations [32]. The lack of a consistent relationship between source country gender inequality and experiencing assault, regional differences, and the importance of the individual-level socio-demographic differences (i.e., education, refugee status) speak to the complexity and potential influence of many forms of discrimination and other unmeasured individual, family and community level factors.

We did not find elevated rates of poor mental health outcomes among those immigrating from countries with high gender inequality. This is in contrast to studies examining variation in gender inequality across countries in Europe that found country-level gender inequality associated with higher levels of depression or depressive symptoms in both men and women [6]. However, our study included only severe outcomes and, as with injuries, our data sources are predicated on care-seeking for ascertainment. Previous research has shown that those of South Asian ethnicity and Black race may underutilize mental health care [33]. Consistent with a prior systematic review [34] and our recent study on self-harm and suicides in immigrants to Ontario [35] we found refugees had increased rates of poor mental health relative to economic immigrants, likely driven by higher pre- and post-migration stressors [36]. The larger and more consistent associations between these individual level factors than with source country factors such as GII or GNI highlights that individual level factors may be more useful in prioritizing preventive interventions. In addition, some of the source country findings in Fig. 1 could help direct further work relevant to some communities of immigrants, ideally in a community participatory framework.

The strengths of our study include using data from Ontario, which has a large population (14+ million), high levels of immigration and diversity of source countries, and availability of administrative databases that enable population-based samples and longitudinal follow-up. Consequently, this study has one of the largest and most diverse samples to investigate assault and adverse mental health outcomes among immigrant females [26]. Including nearly all recent immigrant women to Ontario from all world regions enabled us to examine different regions and countries in relation to one another, in a manner not typical of prior studies examining violence against women, which have often focused on convenience samples of a small number of immigrants from a few select source countries [26, 29].

Our study is not without limitations. First, outcomes were largely based on acute healthcare utilization. Consequently, these outcomes miss less severe mental health problems or assaults and may be influenced by barriers to care seeking and underreporting, which may be more common in some populations of immigrants [33, 37]. Racialized immigrants may not seek care for injuries due to distrust of police given community experience with systemic racism in the justice system [38, 39]. We tried to address under-reporting with sensitivity analyses examining “other injuries”. Second, our assault outcomes were broad and complicate comparison with prior studies on gender-based violence that typically examined self-reported IPV. Third, a lack of data on the perpetrator or circumstances of assaults, as well as important contexts of household and community means we cannot explore important causal pathways and mediators or draw conclusions around the role of gender inequality. While Canadian reports estimate that 57% of police reported violent injuries among women are due to intimate partner or non-spousal family members [40] we do not know whether this is the case across a relatively young and diverse group of immigrants in terms of countries of origin, categories of immigrants and socio-economic status. Fourth, our immigration data include only legal immigrants who migrated to Ontario and obtained permanent residence. Fifth, we did not explore whether there was a differential relationship of individual covariates such as education by GII category or country. Sixth, gender inequality was measured at the country level, precluding assessing regional heterogeneity within countries, which has been shown to be important in studies of male-biased sex ratios in immigrants from India [14]. Lastly, while we were able to include some important socio-demographic characteristics, these were measured at the time of immigration, are not comprehensive, and do not include factors such as language proficiency and employment, which may influence the risk of the outcomes in this study.

## Conclusions

We did not find a consistent relationship between source country gender inequality and acute presentations of mood and anxiety disorder, including self-harm. While there was some association between source country gender inequality and experiencing assault post-migration, it was not consistent and was more related to region of origin, refugee status and other socio-economic factors. Efforts to address gender-based violence in Canadian immigrant communities are unlikely to be meaningfully informed by measures of gender inequality of source countries. They will require community engagement and community-based research to further elucidate the mechanisms by which these reported disparities occur and identify meaningful interventions.

## Supplementary Information

**Additional file 1: Table S1.** Diagnostic codes used to define each outcome. **Fig. S1.** Flowchart of study exclusions. **Fig. S2.** Average GII value during the study recruitment period for each country^a^ of birth. **Table S2.** Number and rate^a^ for other injuries and death^a^. **Table S3.** Poisson regression for association between GII^a^ quartiles and Other injury and death (*N* = 204,124)^b^. **Table S4.** Poisson regression for association between GII^a^ and all outcomes among immigrants age 18–29 at immigration (*N* = 134,338)^b^.

## Data Availability

The dataset from this study is held securely in coded form at ICES. While legal data sharing agreements between ICES and data providers (e.g., healthcare organizations and government) prohibit ICES from making the dataset publicly available, access may be granted to those who meet pre-specified criteria for confidential access, available at www.ices.on.ca/DAS (email: das@ices.on.ca). The full dataset creation plan and underlying analytic code are available from the authors upon request, understanding that the computer programs may rely upon coding templates or macros that are unique to ICES and are therefore either inaccessible or may require modification.

## Abbreviations

GII: Gender Inequality Index
Q: Quartile
IRR: Incident Rate Ratio
CI: Confidence Interval
SA: South Asia(n)
EA: East Asia(n)
SSA: Sub-Saharan Africa(n)
LAC: Latin America and the Caribbean
PY: Person Years
IPV: Intimate Partner Violence
GNI: Gross National Income
ED: Emergency Department
IRCC-PR: Immigration, Refugees and Citizenship Canada Permanent Resident
DAD: Discharge Abstract Database
OMHRS: Ontario Mental Health Reporting System
NACRS: National Ambulatory Care Reporting System
ORG-VSD: Registrar General Vital Statistics Database
ICD: International Classification of Disease
