# Supplementary material for: Association of source country gender inequality with experiencing assault and poor mental health among young female immigrants to Ontario, Canada
Source: BMC Public Health. 2021 Apr 16;21:739. doi: 10.1186/s12889-021-10720-0 (PMC8052772; doi:10.1186/s12889-021-10720-0)
Supplement: Supplementary file 1 — Additional file 1: Table S1. Diagnostic codes used to define each outcome. Fig. S1. Flowchart of study exclusions. Fig. S2. Average GII value during the study recruitment period for each countrya of birth. Table S2. Number and ratea for other injuries and deatha. Table S3. Poisson regression for association between GIIa quartiles and Other injury and death (N = 204,124)b. Table S4. Poisson regression for association between GIIa and all outcomes among immigrants age 18–29 at immigration (N = 134,338)b. [file 12889_2021_10720_MOESM1_ESM.docx]

Supplementary Table 1. Diagnostic codes used to define each outcome

| **Outcome** | **Database** | **Diagnostic Classification** | **Diagnostic Position** | **Diagnostic codes** |
| --- | --- | --- | --- | --- |
| *Composite Mental Health Outcome*  Mood or Anxiety Disorder (MA) Emergency Department (ED) visit | | | | |
| Anxiety disorders | NACRS | ICD 10 | Primary | F40-43, F48.8, F48.9, F93.1, F93.2 |
| Mood disorders | NACRS | ICD 10 | Primary | F30-34, F38, F39, F53.0 |
| MA admission | | | | |
| Anxiety disorders | OMHRS | DSM IV | Primary | 300, 300.0x, 300.2x, 300.3x, 308.3x, 309.0x, 309.24, 309.28, 309.3x, 309.4x, 309.8x, 309.9x.  Provisional diagnosis*: “Anxiety Disorder”, ”Adjustment Disorder” |
| Anxiety disorders | DAD | ICD10 | Primary | F40-43, F48.8, F48.9, F93.1, F93.2 |
| Mood disorders | OMHRS | DSM IV | Primary | Primary Diagnosis: 296.x (all 296 codes), 300.4x, 301.13  Provisional diagnosis*: “Mood Disorder” |
| Mood disorders | DAD | ICD 10 | Primary | F30-34, F38, F39, F53.0 |
| Deliberate Self-Harm (SH) ED visit | NACRS | ICD 10 | Any position | X60-X84 |
| Suicide | ORGD | ICD 9 | Primary position | E950-E959 |
| *Violent Injury* | | | | |
| Assault ED visit | NACRS | ICD 10 | Any position | X85–Y09, Y87.1 |
| Assault admission | DAD | ICD 10 | Any position | X85–Y09, Y87.1 |
| Intimate Partner Violence ED visit | NACRS | ICD 10 | Any position | T74.0-T74.9, Y07.0, Y06.0 |
| Intimate Partner Violence ED visit | DAD | ICD 10 | Any position | T74.0-T74.9, Y07.0, Y06.0 |
| Homicide | ORG-VSD | ICD 10 | Any position | *U01–*U02, X85–Y09, Y87.1 |
| *Other Injury* | NACRS | ICD 10 | Any position | V01-X59, Y85-Y86, Y10-Y34, Y87.2, Y89.9 |
| *All-Cause Mortality* | ORG-VSD, RPDB | NA | NA | NA |

Legend: Abbreviations: NACRS: National Ambulatory Care Reporting System, ICD: International Classification

of Disease, OMHRS: Ontario Mental Health Reporting System, DSM IV: Diagnostic and Statistical Manual of

Mental Disorders 4^th^ Edition IV, DAD: Discharge Abstract Database, ORG-VSD: Ontario Registrar

General Vital Statistics Death, RPDB: Registered Persons Database

Supplementary Figure 1. Flowchart of study exclusions


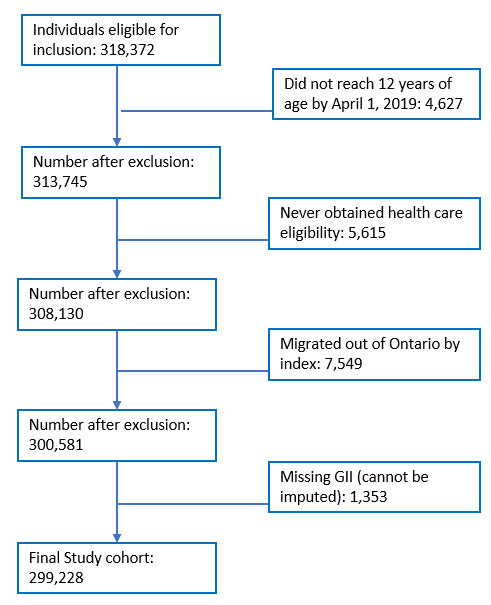


Supplementary Figure 2. Average GII value during the study recruitment period for each country^a^ of birth

Legend:  ^a^For 179 countries present in the sample (13 countries not included because censored due to small cell restrictions); Countries highlighted are those with highest (Yemen) and lowest (Sweden) GII values, the top four source countries, and Canada’s GII value for reference.

Supplementary Table 2. Number and rate^a^ for other injuries and death^a^

|  | Other Injuries | | Death | |
| --- | --- | --- | --- | --- |
|  | N | Rate^a^ | N | Rate^a^ |
| *GII Quartile* |  |  |  |  |
| GII Q1 | 9300 | 146.8 | 88 | 1.4 |
| GII Q2 | 7301 | 192 | 66 | 1.7 |
| GII Q3 | 15164 | 203.3 | 133 | 1.8 |
| GII Q4 | 17970 | 163.3 | 162 | 1.5 |
| *Largest 4 Countries* |  |  |  |  |
| China (GII = 0.20; Q1) | 4034 | 110.4 | 50 | 1.4 |
| Philippines (GII = 0.45; Q3) | 3495 | 158.3 | 32 | 1.4 |
| Pakistan (GII = 0.59; Q4) | 2962 | 136.4 | 23 | 1.1 |
| India (GII = 0.60; Q4) | 6041 | 132.2 | 62 | 1.4 |
| *Age at Immigration (Years)* |  |  |  |  |
| 6-9 | 4290 | 197.6 | 20 | 0.9 |
| 10-13 | 6209 | 203.4 | 30 | 1.0 |
| 14-17 | 6927 | 206.1 | 50 | 1.5 |
| 18-21 | 8337 | 194.3 | 60 | 1.4 |
| 22-25 | 11255 | 163.8 | 118 | 1.7 |
| 26-29 | 12717 | 143.7 | 171 | 1.9 |
| *Immigrant Status* |  |  |  |  |
| Economic | 16887 | 146.9 | 144 | 1.3 |
| Family/other/missing | 22526 | 173.2 | 228 | 1.8 |
| Refugee | 10322 | 252.3 | 77 | 1.9 |
| *Income Quintile* |  |  |  |  |
| Income Quintile 1 (Lowest) | 19525 | 179.7 | 156 | 1.4 |
| Income Quintile 2 | 11480 | 168.3 | 113 | 1.7 |
| Income Quintile 3 | 8613 | 174.5 | 79 | 1.6 |
| Income Quintile 4 | 6240 | 172.3 | 71 | 2 |
| Income Quintile 5 (highest) | 3877 | 164.6 | 30 | 1.3 |
| *Immigration Period* |  |  |  |  |
| 2003-2005 | 17100 | 163.9 | 177 | 1.7 |
| 2006-2008 | 14072 | 175.5 | 123 | 1.5 |
| 2009-2011 | 9582 | 182.5 | 81 | 1.5 |
| 2012-2014 | 6034 | 185.6 | 50 | 1.5 |
| 2015-2017 | 2947 | 179.1 | 18 | 1.1 |
| *Region of Birth* |  |  |  |  |
| East Asia and Pacific | 9460 | 126.7 | 112 | 1.5 |
| Europe and Central Asia | 6750 | 210.4 | 62 | 1.9 |
| Latin America and the Caribbean | 7971 | 252.6 | X | X |
| Middle East and North Africa | 6517 | 197.7 | 51 | 1.5 |
| North America | 1597 | 237.5 | X | X |
| South Asia | 13333 | 149.9 | 110 | 1.2 |
| Sub-Saharan Africa | 4107 | 215.6 | 53 | 2.8 |
| *GNI Economy Categorization* |  |  |  |  |
| Low-income | 11406 | 161.5 | 117 | 1.7 |
| Lower-middle-income | 22334 | 163.9 | 202 | 1.5 |
| Upper-middle-income | 9507 | 203.6 | 86 | 1.8 |
| High-income | 6488 | 200.4 | 44 | 1.4 |
| *Marital Status (Immigrants age 18-29 at immigration)* |  |  |  |  |
| Married | 12436 | 180 | 120 | 1.7 |
| Separated/widowed/divorced | 350 | 277.9 | X | X |
| Single | 19517 | 150.5 | X | X |
| *Education (Immigrants age 18-29 at immigration)* |  |  |  |  |
| Bachelors or higher | 9887 | 129.7 | 117 | 1.5 |
| Postgraduate less than Bachelors | 6464 | 166.4 | 75 | 1.9 |
| Secondary or less | 15754 | 188 | 156 | 1.9 |

Legend: X = censored due to small cell restrictions; ^a^per 10,000 Person Years (PYs); ^a^Supplementary Table 2 uses full sample before stratified random sampling is applied

Supplementary Table 3. Poisson regression for association between GII^a^ quartiles and Other injury and death (N=204,124)^b^

|  | Other Injury | | | Death | | |
| --- | --- | --- | --- | --- | --- | --- |
|  | IRR | 95%LCL | 95%UCL | IRR | 95%LCL | 95%UCL |
| *GII Quartile (Ref: GII Q1)^a^* |  |  |  |  |  |  |
| GII Q2 | 0.98 | 0.83 | 1.15 | 1.15 | 0.70 | 1.88 |
| GII Q3 | 1.09 | 0.92 | 1.30 | 1.34 | 0.78 | 2.29 |
| GII Q4 | 1.06 | 0.86 | 1.30 | 1.19 | 0.69 | 2.05 |
| *Largest 4 Countries* |  |  |  |  |  |  |
| China | 0.61 | 0.52 | 0.71 | 1.26 | 0.78 | 2.04 |
| Philippines | 0.85 | 0.78 | 0.92 | 1.13 | 0.87 | 1.46 |
| Pakistan | 0.75 | 0.69 | 0.82 | 0.44 | 0.32 | 0.60 |
| India | 0.77 | 0.71 | 0.84 | 0.63 | 0.47 | 0.84 |
| *Age (Years) (Ref: 26-29)* |  |  |  |  |  |  |
| 6-9 | 1.38 | 1.27 | 1.48 | 0.45 | 0.28 | 0.71 |
| 10-13 | 1.36 | 1.27 | 1.44 | 0.50 | 0.32 | 0.78 |
| 14-17 | 1.35 | 1.28 | 1.43 | 0.69 | 0.43 | 1.09 |
| 18-21 | 1.26 | 1.19 | 1.33 | 0.74 | 0.52 | 1.07 |
| 22-25 | 1.08 | 1.04 | 1.12 | 0.88 | 0.68 | 1.15 |
| *Income Quintile (Ref: Quintile* *1)* |  |  |  |  |  |  |
| Income Quintile 2 | 0.98 | 0.95 | 1.01 | 1.02 | 0.80 | 1.31 |
| Income Quintile 3 | 1.02 | 0.97 | 1.06 | 1.17 | 0.84 | 1.63 |
| Income Quintile 4 | 0.98 | 0.93 | 1.03 | 1.31 | 0.98 | 1.75 |
| Income Quintile 5 (Highest) | 0.94 | 0.87 | 1.01 | 0.87 | 0.53 | 1.43 |
| *Gross National Income (GNI) Categorization (Ref: Low income)* |  |  |  |  |  |  |
| Lower-middle-income | 1.12 | 1.00 | 1.25 | 0.75 | 0.53 | 1.07 |
| Upper-middle-income | 1.27 | 1.08 | 1.50 | 1.07 | 0.69 | 1.67 |
| High-income | 1.25 | 1.01 | 1.55 | 0.87 | 0.46 | 1.63 |
| *Immigration Status (Ref: Economic)* |  |  |  |  |  |  |
| Refugee | 1.58 | 1.47 | 1.70 | 1.38 | 1.03 | 1.84 |
| Family | 1.26 | 1.17 | 1.35 | 1.11 | 0.87 | 1.41 |
| *Immigration Period (Ref: 2003-2005)* |  |  |  |  |  |  |
| 2006-2008 | 1.02 | 0.98 | 1.06 | 0.85 | 0.66 | 1.09 |
| 2009-2011 | 1.05 | 0.99 | 1.11 | 0.82 | 0.59 | 1.13 |
| 2012-2014 | 1.07 | 0.99 | 1.15 | 0.84 | 0.59 | 1.19 |
| 2015-2017 | 1.03 | 0.93 | 1.14 | 0.44 | 0.22 | 0.90 |

Legend: ^a^GII = Gender Inequality Index; ^b^Experiencing assault and death are modeled in separate regression models.

Supplemental Table 4. Poisson regression for association between GII^a^ and all outcomes among immigrants age 18-29 at immigration(N=134,338)^b^

|  | Experiencing Assault | | | Mental Health Composite Outcome | | |
| --- | --- | --- | --- | --- | --- | --- |
|  | IRR | 95%LCL | 95%UCL | IRR | 95%LCL | 95%UCL |
| *GII^b^ Quartile (Ref: GII Q1)* |  |  |  |  |  |  |
| GII Q2 | 1.41 | 1.05 | 1.89 | 0.84 | 0.67 | 1.06 |
| GII Q3 | 1.35 | 0.90 | 2.03 | 0.95 | 0.73 | 1.24 |
| GII Q4 | 1.59 | 1.04 | 2.43 | 1.11 | 0.81 | 1.53 |
| *Largest 4 Countries* |  |  |  |  |  |  |
| China | 1.41 | 1.03 | 1.92 | 0.41 | 0.33 | 0.52 |
| Philippines | 1.17 | 0.88 | 1.55 | 0.70 | 0.56 | 0.87 |
| Pakistan | 0.47 | 0.38 | 0.57 | 0.73 | 0.60 | 0.88 |
| India | 0.52 | 0.42 | 0.65 | 0.67 | 0.56 | 0.81 |
| *Age (Years) (Ref: 26-29)* |  |  |  |  |  |  |
| 18-21 | 1.30 | 1.05 | 1.61 | 1.25 | 1.10 | 1.42 |
| 22-25 | 1.04 | 0.87 | 1.24 | 1.09 | 0.98 | 1.22 |
| *Income Quintile (Ref: Quintile 1)* |  |  |  |  |  |  |
| Income Quintile 2 | 0.91 | 0.78 | 1.05 | 0.93 | 0.85 | 1.03 |
| Income Quintile 3 | 0.81 | 0.68 | 0.97 | 0.98 | 0.86 | 1.11 |
| Income Quintile 4 | 0.82 | 0.67 | 1.00 | 0.91 | 0.81 | 1.02 |
| Income Quintile 5 | 0.86 | 0.66 | 1.12 | 0.95 | 0.79 | 1.13 |
| *Gross National Income (GNI) Categorization (Ref: Low income)* |  |  |  |  |  |  |
| Lower-middle-income | 0.94 | 0.68 | 1.30 | 1.09 | 0.87 | 1.36 |
| Upper-middle-income | 1.23 | 0.84 | 1.80 | 1.28 | 0.97 | 1.68 |
| High-income | 1.23 | 0.81 | 1.85 | 1.25 | 0.83 | 1.88 |
| *Immigrant Status (Ref: Economic)* |  |  |  |  |  |  |
| Refugee | 2.51 | 1.94 | 3.25 | 1.76 | 1.47 | 2.11 |
| Family | 1.94 | 1.58 | 2.38 | 1.39 | 1.19 | 1.63 |
| *Immigration Period (Ref: 2003-2005)* |  |  |  |  |  |  |
| 2006-2008 | 1.07 | 0.90 | 1.27 | 0.96 | 0.86 | 1.07 |
| 2009-2011 | 1.23 | 1.02 | 1.49 | 0.97 | 0.84 | 1.10 |
| 2012-2014 | 1.23 | 0.96 | 1.58 | 0.97 | 0.86 | 1.10 |
| 2015-2017 | 1.14 | 0.87 | 1.51 | 1.15 | 0.98 | 1.35 |
| *Marital Status (Ref: Single)* |  |  |  |  |  |  |
| Married | 0.71 | 0.62 | 0.82 | 0.89 | 0.80 | 0.99 |
| Separated/divorced/widowed | 1.09 | 0.76 | 1.56 | 1.43 | 0.95 | 2.14 |
| *Education (Ref: Secondary or less)* |  |  |  |  |  |  |
| Postgraduate less than Bachelors | 0.95 | 0.77 | 1.17 | 0.90 | 0.79 | 1.01 |
| Bachelors or higher | 0.64 | 0.51 | 0.80 | 0.69 | 0.60 | 0.80 |
|  | Other Injury | | | Death | | |
|  | IRR | 95%LCL | 95%UCL | IRR | 95%LCL | 95%UCL |
| *GII Quartile (Ref: GII Q1)* |  |  |  |  |  |  |
| GII Q2 | 0.99 | 0.86 | 1.15 | 0.93 | 0.58 | 1.48 |
| GII Q3 | 1.14 | 0.96 | 1.35 | 1.29 | 0.72 | 2.31 |
| GII Q4 | 1.17 | 0.94 | 1.47 | 1.28 | 0.67 | 2.45 |
| *Largest 4 Countries* |  |  |  |  |  |  |
| China | 0.66 | 0.57 | 0.77 | 1.32 | 0.79 | 2.19 |
| Philippines | 0.88 | 0.81 | 0.96 | 1.22 | 0.91 | 1.63 |
| Pakistan | 0.76 | 0.69 | 0.83 | 0.48 | 0.35 | 0.66 |
| India | 0.79 | 0.73 | 0.87 | 0.68 | 0.50 | 0.91 |
| *Age (Years) (Ref: 26-29)* |  |  |  |  |  |  |
| 18-21 | 1.11 | 1.04 | 1.18 | 0.57 | 0.35 | 0.92 |
| 22-25 | 1.03 | 0.99 | 1.07 | 0.80 | 0.61 | 1.06 |
| *Income Quintile (Ref: Quintile 1)* |  |  |  |  |  |  |
| Income Quintile 2 | 1.00 | 0.96 | 1.03 | 0.98 | 0.76 | 1.27 |
| Income Quintile 3 | 1.01 | 0.95 | 1.06 | 1.05 | 0.70 | 1.56 |
| Income Quintile 4 | 0.99 | 0.93 | 1.05 | 1.41 | 1.01 | 1.96 |
| Income Quintile 5 | 0.92 | 0.84 | 1.00 | 0.90 | 0.51 | 1.58 |
| *Gross National Income (GNI) Categorization (Ref: Low income)* |  |  |  |  |  |  |
| Lower-middle-income | 1.14 | 1.01 | 1.29 | 0.69 | 0.46 | 1.03 |
| Upper-middle-income | 1.32 | 1.10 | 1.59 | 1.12 | 0.68 | 1.82 |
| High-income | 1.28 | 0.96 | 1.70 | 0.92 | 0.47 | 1.79 |
| *Immigrant Status (Ref: Economic)* |  |  |  |  |  |  |
| Refugee | 1.60 | 1.49 | 1.73 | 1.34 | 0.98 | 1.84 |
| Family | 1.27 | 1.18 | 1.37 | 0.98 | 0.72 | 1.32 |
| *Immigration Period (Ref: 2003-2005)* |  |  |  |  |  |  |
| 2006-2008 | 1.03 | 0.98 | 1.08 | 0.86 | 0.63 | 1.16 |
| 2009-2011 | 1.04 | 0.97 | 1.11 | 0.80 | 0.56 | 1.16 |
| 2012-2014 | 1.06 | 0.97 | 1.15 | 0.74 | 0.47 | 1.17 |
| 2015-2017 | 1.07 | 0.97 | 1.19 | 0.46 | 0.22 | 0.92 |
| *Marital Status (Ref: Single)* |  |  |  |  |  |  |
| Married | 0.90 | 0.86 | 0.94 | 0.85 | 0.64 | 1.15 |
| Separated/divorced/widowed | 1.23 | 1.07 | 1.41 | 0.28 | 0.04 | 2.19 |
| *Education (Ref: Secondary or less)* |  |  |  |  |  |  |
| Postgraduate less than Bachelors | 0.98 | 0.93 | 1.03 | 0.94 | 0.68 | 1.31 |
| Bachelors or higher | 0.84 | 0.78 | 0.90 | 0.65 | 0.45 | 0.95 |

Legend: ^a^GII = Gender Inequality Index; Legend: ^b^Each outcome is modeled in a separate regression model.
